# Paralysis from Arsenic and Treatment by Firing

**Published:** 1847-12

**Authors:** 


*Paralysis from Arsenic and treatment by firing.*—In the *Dublin Gazette*, Dr. Corrigan gives the history of a case of the above disease, of which the following is an outline; and he subsequently explains and remarks on the form of treatment which he denominates "firing" or scorching, rather, as distinguished from actual cauterization.

"A laborer was admitted under Dr. Corrigan's care, Dec. 7th, 1845, laboring under paralysis of upper and lower extremities. The paralysis was greater in the lower extremities, which were unable to support the weight of his body. The muscles of his thighs and legs were flaccid and wasted, but sensibility not impared. His intellect is unimpaired; the pulse is quick and rather compressible, the bowels regular, and the appetite good.

"He attributes the present attack of paralysis to a dose of arsenic which he took by mistake for flour. On having taken it, he was brought to an hospital in Liverpool, and the stomach-pump used. Immediately afterwards, he began to feel disagreeable prickling sensations in his hands and in the soles of the feet. He soon began to totter in his walk; his limbs then became unable to support the weight of his body, and in this state he was admitted into the hospital.

"Five grains of blue pill were given three times a day, until the mouth became sore, for a week.

17th.—No improvement; the firing-iron was applied along the spine and over the thighs and legs.

"20.—He has regained considerable power over the arms, but not over the lower limbs.

"The firing is applied every day along the spine, thighs, and legs.

"24.—He is now able to walk, firing to be continued daily; one sixteenth of a grain of strychnine three times a day.

"January 5th, 1847.—Limbs rapidly regaining their strength. On the 8th inst. all treatment was discontinued. He was retained in the Hospital until the 23d instant, lest any relapse should occur.

He occasionally called at the hospital, and was, at the date of the last report, February 25th, in the possession of perfect strength."

Dr. Corrigan then remarks:—

"I wish to point out the mode of using this application, and the reason for preferring it to other counter-irritants. The iron used is portable, consisting of a thick iron-wire shank, about two inches long, inserted in a small wooden handle, having on its extremity, which is slightly curved, a disc or button of iron, a quarter of an inch thick, and half an inch in diameter, the whole instrument being six inches in length. The face of the disc for application is flat. This, trifling as it may seem, must be attended to. In the French cauterizing irons, the bottoms for cauterizing are spherical, and the consequence is, that they must either be pressed long and deeply into the skin, to bring them in contact with an extent of surface equal to their diameter, or they can be made only to touch at a single point. The only other portion of apparatus required is a small brass spirit-lamp. To use the instrument, it is only necessary to hold the button of the instrument over the flame, keeping the forefinger of the hand holding the instrument at the distance of about half an inch from the button. As soon as the finger feels uncomfortably hot, the instrument is ready for use, and the time required for heating it to this degree is only about a quarter of a minute. It is applied as quickly as possible, the skin being tipped successively, at intervals of half an inch, over the whole affected part, as lightly and as rapidly as possible, always taking care to bring the flat surface of the disc fairly in contact with the skin. In this way the process of firing a whole limb, or the loins making about one hundred applications, does not occupy a minute, and the one heating by the lamp suffices. You can ascertain at once whether the heat be sufficient. If you look sideways at the spots as you touch them, you will observe that each spot the iron has touched immediately becomes of a glistening white, much whiter than the surrounding skin. In the course of a quarter of an hour, sometimes of a very few minutes, the whole skin becomes of a bright red, and the patient feels a glow of heat over the part. The iron is never rendered red hot. It is indeed very little hotter than boiling water, and he never makes an eschar with it, and very rarely indeed raises a blister. There are merely seen upon the skin next day, a number of circular red marks, the cuticle not being raised, and the surface being ready, if required, to receive a fresh application, and what is of no trifling consequence, where such an extent of counter-irritation has been used, there being no discharging surface to interfere with the motion of the limb, or the comforts of the patient. In most cases, the patient is quite unconscious of what has been done. In the case of a medical friend of the author, he could not ever guess what the application was; he merely knew that a not disagreeable smarting or heating sensation was suddenly produced but could not say how, until he saw the instrument. The thickness of the disc or button of the instrument is not a matter of indifference. If it be thinner than the measure given, it will cool too rapidly; if it be thicker, it will take too long a time in heating."

Dr. Corrigan goes on to say—

"I can certainly recommend its application in the way here directed, as one of the most useful of our counter-irritants; it can be applied so rapidly, so extensively, and, as I have already said, without even the patient knowing, very often, what has been done. Its superiority over blisters may also be owing to the suddenness of the impression produced. The effect is often as instantaneous as the application. My friend Dr. Mitchell, Master of the Victoria Lying-in Hospital, consulted me some months since for a severe attack of lumbago. I applied the firing, and he was in one minute quite free from pain. He has since used it himself extensively in practice, and, I believe, he will shortly publish the results of his observations."

To this succeeds the mention of several equally successful cases quite sufficient to justify a trial of the remedy.—*London Lancet.*

